# Multi-Task Scenario Encrypted Traffic Classification and Parameter Analysis

**DOI:** 10.3390/s24103078

**Published:** 2024-05-12

**Authors:** Guanyu Wang, Yijun Gu

**Affiliations:** College of Information and Cyber Security, People’s Public Security University of China, Beijing 100038, China; gyc0016@163.com

**Keywords:** encrypted traffic, network management, interpretability analysis, fine-tuning

## Abstract

The widespread use of encrypted traffic poses challenges to network management and network security. Traditional machine learning-based methods for encrypted traffic classification no longer meet the demands of management and security. The application of deep learning technology in encrypted traffic classification significantly improves the accuracy of models. This study focuses primarily on encrypted traffic classification in the fields of network analysis and network security. To address the shortcomings of existing deep learning-based encrypted traffic classification methods in terms of computational memory consumption and interpretability, we introduce a Parameter-Efficient Fine-Tuning method for efficiently tuning the parameters of an encrypted traffic classification model. Experimentation is conducted on various classification scenarios, including Tor traffic service classification and malicious traffic classification, using multiple public datasets. Fair comparisons are made with state-of-the-art deep learning model architectures. The results indicate that the proposed method significantly reduces the scale of fine-tuning parameters and computational resource usage while achieving performance comparable to that of the existing best models. Furthermore, we interpret the learning mechanism of encrypted traffic representation in the pre-training model by analyzing the parameters and structure of the model. This comparison validates the hypothesis that the model exhibits hierarchical structure, clear organization, and distinct features.

## 1. Introduction

In network management and cybersecurity domains, network traffic classification plays an integral role. Traffic classification refers to the process of identifying and distinguishing different categories of network traffic according to specific requirements and designs, enabling further analysis. Accurate network traffic classification can help us obtain an overall perception of network bandwidth, capture different network usage habits, and assess network security. Internet Service Providers (ISPs) optimize network resources and enhance network management through traffic classification, while security departments leverage traffic analysis to monitor network security states, identifying and responding to malicious network attacks. However, the widespread use of traffic encryption technologies such as Transport Layer Security (TLS) presents significant challenges to network traffic classification [1]. Encrypted traffic, where plaintext payloads are encrypted and transmitted as ciphertext, can only be decrypted by the sender and the receiver, making it difficult for third parties to interpret. Traditional traffic analysis methods, which rely on extracting valuable information from plaintext payloads (such as port-based methods, Deep Packet Inspection (DPI) methods, and statistical methods), may become ineffective. This encryption prevents network administrators from directly extracting useful plaintext information from network traffic, complicates management tasks, and creates convenient channels for malicious actors to transmit malicious traffic, increasing network security risks [2,3].

In this complex scenario, encrypted traffic classification methods based on feature engineering extract statistically designed features and classify them using classical machine learning [4,5,6,7] or deep learning [8,9]. This approach differs from traditional methods as they do not solely rely on plaintext information such as ports, resulting in better classification performance and ease of deployment. However, performance depends on the comprehensiveness and effectiveness of manually selected features [10]. Alternatively, representation learning-based methods for encrypted traffic classification employ deep learning models to automatically extract and process representations from encrypted traffic [2,10,11,12,13,14]. This approach has become a high-performance algorithmic solution to the problem of encrypted traffic classification [15,16]. While these methods can improve classification performance, they also expose the immaturity of applying deep learning to this problem and are constrained by the limitations inherent in deep learning itself. Improper design may result in biased conclusions or exaggerated classification outcomes. Neglecting the alignment between deep learning methods and the challenges in this domain prevents designers from fully harnessing the potential of deep learning [17]. Unlike natural language and images, encrypted byte streams are difficult for humans to understand. The relevance of the loaded bytes, coupled with the complexity of the deep learning model itself, makes the problem of poor interpretability even more pronounced.

Among the many deep learning frameworks, pre-training models have been extensively applied and have shown exceptional performance in fields such as Natural Language Processing (NLP) and Computer Vision (CV) [14]. In the increasingly complex network environment, pre-training model-based encrypted traffic classification methods outperform other deep learning architectures. Pre-training models first undergo training with a large volume of unlabeled data to pre-train the initial model, followed by fine-tuning with a small amount of labeled data from downstream tasks. The fine-tuned model can then be directly applied to well-designed tasks [18]. However, traditional pre-training methods that adjust all parameters of the model lead to increasingly expensive training costs as the model size and number of downstream tasks grow.

The feature engineering-based encrypted traffic classification methods and the representation learning-based methods demonstrate that it is possible to classify traffic in a fine-grained manner. Statistical representation, plaintext information, and features present in the original payloads are discriminative enough to make each class easily separated from one another [19]. We therefore focus on designing a fine-grained encrypted traffic classification method with broad applicability and stable results in a generic network environment. We aim to implement encrypted traffic functional classification methods under limited conditions. The application scenarios for encrypted traffic classification include four types: (I) the network analytics domain, (II) the network security domain, (III) the user privacy domain, and (IV) the domain of network functions in middleboxes [3]. Our research primarily focuses on two subdomains within Scenarios (I) and (II): application identification, network intrusion detection, and malware detection, particularly emphasizing application identification in different network environments. Specifically, we aim to design a representation learning framework that can be trained in different scenarios to achieve encrypted traffic functional classification. For example, distinguishing different services (VPN Email, Email, VPN Chat, Chat, etc.) and applications (Netflix, P2P, SCP, etc.) in VPN traffic, distinguishing normal traffic (BitTorrent, Facetime, FTP, etc.) from traffic generated by malware (Cridex, Geodo, etc.) in mixed traffic, and distinguishing different attack traffic in the IoT environment (DDoS ACK Fragmentation, ARP Spoofing, XSS, etc.).

In this paper, we propose the Parameter-Efficient Fine-Tuning (PEFT)-based Encrypted Traffic Representation Learning Method (PETReLM). We develop suitable data preprocessing methods based on the characteristics of encrypted traffic and use pre-training models for representation learning to accomplish encrypted traffic classification tasks.

Our contributions are as follows:

- We propose a novel packet-level encrypted traffic classification method based on pre-trained models and PEFT methods. We introduce the Low-Rank Adaptation (LoRA) [20] method into encrypted traffic classification, enhancing pre-training and fine-tuning methods to adapt to encrypted traffic classification tasks. Our method can learn different types of traffic representations in different scenarios with wide applicability and also improve the efficiency of parameter utilization.
- We validate the existence of comprehensive traffic representation information in individual packets and demonstrate the feasibility of classifying traffic for specific scenarios based on these representations. We also discuss the limitations of packet-level encrypted traffic classification.
- Based on singular values and vectors, we compare the matrix parameters of the fine-tuned model with those of the pre-trained model to analyze the model’s representation learning mechanism and fine-tuning principles.

The rest of the paper is structured as follows: Section 2 introduces related work on encrypted traffic classification and PEFT. Section 3 describes our proposed method for encrypted traffic classification. Section 4 presents our experimental settings and results. In Section 5, we analyze the mechanism of encrypted traffic representation extraction and fine-tuning. In Section 6, we discuss the limitations of our method and conclude the paper.

## 2. Related Works

In this section, we review the related research in the fields of encrypted traffic classification and PEFT, discussing the advantages and limitations of these methods.

### 2.1. Encrypted Traffic Classification

The methods of encrypted traffic classification mainly include feature engineering-based approaches and representation learning-based methods. The objective is to classify encrypted traffic according to predefined criteria by learning statistical features, payload features, etc., of the encrypted traffic. Table 1 reports relevant work in these domains and summarizes the fundamental aspects of the methodologies employed.

#### 2.1.1. Feature Engineering-Based Methods

Feature engineering-based methods involve extracting manually designed traffic features and classifying encrypted traffic using classic machine learning or deep learning. Commonly effective features include basic features, time-series features, statistical features, and multi-protocol payload characteristics [21]. Designers produce features based on experience, screen features by comparing their information content and redundancy, and provide these features as input to the designed model. Common classification granularities include data flows (unidirectional or bidirectional), TCP connections, etc.

FlowPrint [4] utilizes the temporal correlation of traffic communication destination addresses to discover different patterns in network traffic, creating a fingerprint library for network traffic. APPScanner [5] employs manually selected traffic statistical features to identify mobile applications, exploring the extent to which mobile app fingerprints can be constructed and assessing the robustness of the app fingerprint framework. Conti et al. [6] generate different time-series cumulative graphs based on varied network behaviors and learn the associations of network traffic cumulative graphs related to specific behaviors. Shen et al. [7] propose constructing application fingerprints by merging the application attribute bigrams into the second-order homogeneous Markov chains, where the attribute bigram comprises certificate packet lengths and the size of the first application data in encrypted sessions. Yu et al. [8] extract traffic statistical features and certificate features from the TLS handshake to the verification phase, extend traffic features into higher dimensions, and introduce hierarchical clustering to reduce data computation. Shen et al. [9] use traffic packets to build the traffic interaction graph and then employ Graph Neural Networks (GNNs) to achieve decentralized APP recognition.

The aforementioned methods lack flexibility and adaptability in the realistic mobile context. Feature engineering relies on task characteristics and expert experience. Manually selected features may not fully capture the characteristics of traffic. This selection process, coupled with method usage, imposes certain limitations on the applicability of classification methods.

#### 2.1.2. Representation Learning-Based Methods

Representation learning-based methods avoid manually designing traffic features and allow for deep learning frameworks to automatically extract representations from raw encrypted byte streams in an end-to-end manner. Typically, these methods focus on classifying at the granularity of data flows (unidirectional or bidirectional), packets, TCP connections, and traffic bursts.

Wang et al. [11] use a One-Dimensional Convolutional Neural Network (1D-CNN) for end-to-end encrypted traffic classification. They transform traffic into two-dimensional images by truncating traffic payloads to a fixed length and converting bytes into image pixels, subsequently using 1D-CNN to learn image representations. Lotfollahi et al. [10] introduce Deep Packet architecture employing Stacked Auto-Encoders (SAE) [22] and a 1D-CNN to handle both traffic representation and application identification tasks. For learning temporal dimension representations, TSCRNN [2] proposes using 1D-CNN to extract spatial features of encrypted traffic, followed by stacked BiLTSM [23] to extract temporal features based on these low-dimensional feature mappings. Jiang et al. [12] use BiLSTM and TextCNN [24] to capture local features and temporal relationship of traffic, employing a multi-head attention mechanism to select important features and reduce the impact of noisy features. PERT [13] devises a novel method to convert encrypted traffic payloads into byte streams, using the A Lite BERT (ALBERT) model [25] for packet-level traffic representation learning, learning the contextual distribution of unlabeled payload bytes, and then reusing the pre-trained model for data stream-level fine-tuning. ET-BERT [14] designs an encrypted traffic representation model based on Bidirectional Encoder Representation from Transformers (BERT) [18] and pre-training tasks more suitable for encrypted traffic, achieving significant improvements in generalization ability and performing well on multiple datasets.

Methods that take raw input data as input enable models to automatically extract representations from the raw data, reducing the overall reliance on human expertise. While current methods achieve good results, deep learning models selected by these representation learning-based methods face certain constraints. The performance of the methods is influenced by preprocessing methods for raw traffic, the adaptability of deep learning models to encrypted traffic classification tasks, and the inherent limitations of deep learning itself, such as slow convergence, high computational resource usage, and poor interpretability.

### 2.2. PEFT

PEFT aims to enhance the performance of pre-training models on new tasks by reducing the number of fine-tuning parameters and computational complexity, thereby alleviating the high training costs of large pre-training models. PEFT methods can overcome catastrophic forgetting [26] and exhibit excellent robustness in out-of-distribution evaluations [27].

Houlsby et al. [28] design the Adapter module consisting of two feedforward projection matrices and a nonlinear layer embedded within the Transformer [29] structure. During training, Transformer parameters are frozen, and only the newly added Adapter module parameters are adjusted. The Adapter achieves results close to full fine-tuning with just 3.6% of the original model’s parameter size. Lin et al. [27] construct a set of prompt tokens as Prefix connected to the left side of each attention layer input in Transformer, keeping model parameters unchanged during fine-tuning and updating only the Prefix part. The Prefix Tuning approach has achieved good results in various language models. Liu et al. [30] develop the IA3 method, which scales activations by learned vectors in attention layers and the Feedforward Neural Network (FNN), achieving stronger performance than full model fine-tuning. Liu et al. [31] propose converting prompts into learnable embedding layers and processing prompt embedding layers with MLP+LSTM, enhancing BERT’s performance on few-sample tasks, and significantly reducing the need for prompt engineering. Hu et al. [20] design the LoRA method based on the intrinsic “low rank” of parameter update matrices. LoRA maintains the pre-trained model parameters unchanged and uses two low-rank matrices to replace weight update matrices, avoiding inference delay issues introduced by inserting other modules.

The shortcoming of PEFT is that it sacrifices a portion of the model’s performance and fails to establish a connection with the pre-trained model, leaving room for improvement in interpretability.

## 3. Our Methods

In this section, we present the overall framework and implementation details of the PETReLM. The PETReLM is designed to extract representations of encrypted traffic and classify traffic based on traffic representations. Our open-source code is hosted at the following GitHub repository: https://github.com/ssy198/PETReLM.

### 3.1. Architectural Overview

**Figure 1:**
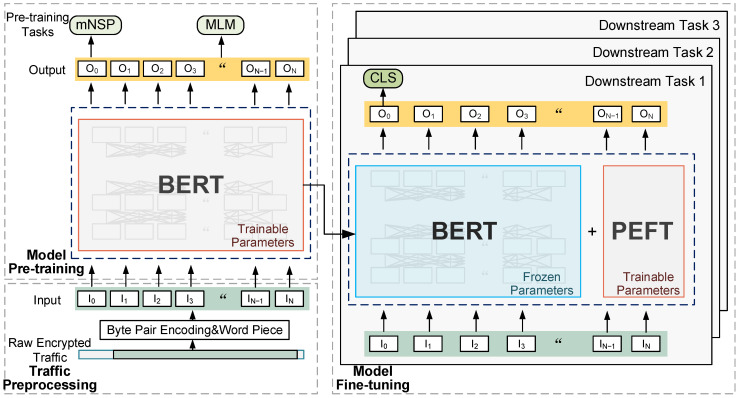
Overview of model framework.

We propose the overall framework of the PETReLM as shown in Figure 1. We select BERT [18] as the foundational model architecture for pre-training and fine-tuning, and the approach consists of three main stages:

(1) Traffic Preprocessing: This stage involves trimming and transforming raw encrypted traffic data. We tailor traffic packets by removing irrelevant information and the convert tailored traffic into a format suitable for the model.
(2) Model Pre-training: This stage employs a Masked Language Model (MLM) and a modified Next Sentence Prediction (mNSP) task as pre-training tasks for the model to learn general representations of traffic. This enables the model to initially extract basic general representations.
(3) Model Fine-tuning: In this stage, the pre-trained model parameters are reused for downstream classification task training. The pre-trained model parameters are frozen, and the newly embedded PEFT module and added fully connected layer classifier parameters are fine-tuned. This enables the model to learn task-specific representations and achieve encrypted traffic classification in specific scenarios.

### 3.2. Preprocessing

The preprocessing of traffic involves traffic trimming and traffic transformation. Traffic trimming refers to appropriately trimming the original traffic to reduce interference from noise information. Traffic transformation refers to converting the trimmed traffic into a data format suitable for model input. We classify encrypted traffic in a packet level, with specific rationale detailed in Section 4.1.

We analyze packet structure based on the TCP/IP protocol suite and discuss which part should be retained or discarded. Because the data link layer (L2) header and trailer only contain essential control information for communication between adjacent hosts, which is not very helpful for encrypted traffic classification, they should be removed. The network layer (L3) IP packet header mainly includes communication addresses (IP addresses) within the packet-switched network. While the server’s IP address in normal traffic is valuable for encrypted traffic classification, considering that VPNs, malware, etc., may obscure actual IP addresses and the complexity of translating IP addresses into meaningful information for deep learning models, we remove the network layer header information. The transport layer (L4) is a crucial part of a packet, consisting of the TCP and UDP protocols. Most services are designed based on these protocols. The transport layer header includes information such as ports and session control. Ports indicate information about processes, and if the port is a well-known port number, the service category can be directly determined. Although ports may be obfuscated in some malicious traffic scenarios, the transport layer header is an important reference for host process communication behavior. We believe that retaining information from the transport layer header yields greater benefits than the information loss from discarding it, so we keep the relevant information from the transport layer. Finally, the application layer (L5) is a crucial reference for encrypted traffic classification and is retained. In summary, to retain efficient representations that enable the separation of different categories for downstream tasks, we discard network layer headers and retain relevant information from the transport layer and the application layer for further processing.

The length of encrypted traffic payload is not fixed, and we also need to preserve the transportation layer header information and part of the application layer payload byte stream by the truncation operation. This step reduces the dimensions of the input data and improves the efficiency of model training and inference. Details regarding the truncation operation are discussed in Section 4.2.

**Figure 2:**
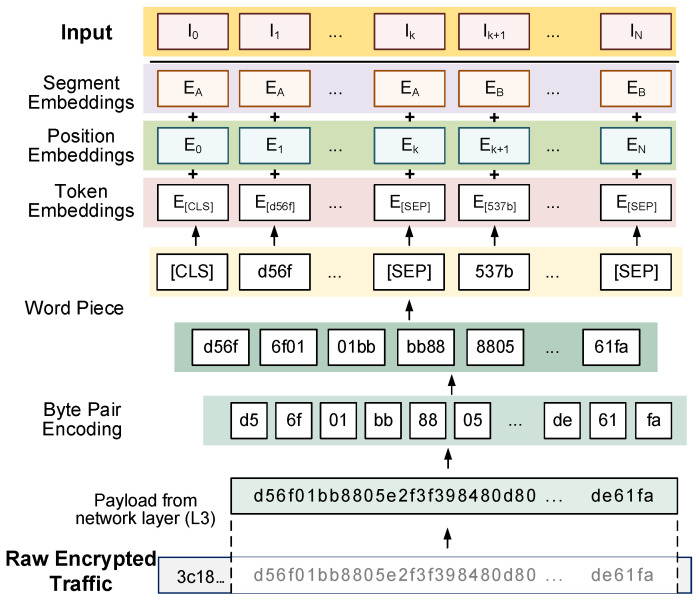
Encrypted traffic preprocessing.

Encrypted traffic is a distinct data type separate from natural language or images. However, from an abstract perspective, traffic data can be considered sequential data. Therefore, we employ sequence data preprocessing methods to handle trimmed traffic payloads. To transform the byte stream of payloads into a sequence of basic character units akin to natural language, we employ a Byte-Pair Encoding method [13]. It concatenates two adjacent bytes to form a basic character unit (0000—ffff), turning the byte stream into a string of byte pairs. Subsequently, the Word Piece algorithm [32] is used to tokenize these byte pair strings. For subsequent task processing, special tokens [CLS], [SEP], [PAD], and [MASK] are added to the dictionary generated by the Word Piece algorithm. Each token sequence begins with [CLS], representing the hidden layer state output for the final classification task. [SEP] marks the end of a sub-sequence, [PAD] is used for padding sequences to reach a minimum length, and [MASK] is used to obscure existing tokens for pre-training task MLM. The token embedding transformed by the Word Piece algorithm is summed with the segment and position embeddings to obtain the input sequence. Figure 2 illustrates the process of traffic preprocessing.

### 3.3. Pre-Training

Pre-training leverages unlabeled data to train the model to learn general representations of encrypted traffic. Plaintext payloads are transformed into unintelligible ciphertext through encryption algorithms. Cryptographic implementation of encryption algorithms exhibits a certain degree of non-complete randomness [33], indicating a high information content and high uncertainty in the ciphertext. Encryption algorithms (e.g., AES, etc.) mix bytes from input plaintext blocks, enhancing the entropy of the ciphertext and also increasing the correlation between bytes within the blocks. Although the ciphertext itself appears random, there are still some abstract representations that can be learned by neural networks, such as the frequency of occurrence of certain bytes and the relationships between specific bytes. Therefore, we directly introduce BERT’s original pretraining task, MLM, and improve the original NSP method to adapt to the byte-level representations of the encrypted traffic.

The model’s core structure is a BERT base [18], consisting of 12 Transformer [29] Encoder modules. Each Encoder’s input and output vectors correspond one to one and maintain consistent dimensions. Within each Encoder module, there are two sequential sub-layers: the first is a multi-head self-attention mechanism, and the second is an FNN. Both sub-layers employ residual connections followed by layer normalization.

To learn the contextual relationships of token embeddings, the MLM task randomly masks part of the input tokens, and the output hidden layer vectors corresponding to the masked tokens are computed in a fully connected layer to predict the actual tokens. A total of 15% of the tokens in the input sequence are masked, with 80% replaced by [MASK], 10% replaced by random tokens, and 10% unchanged. The negative log-likelihood function is used as the loss function for this task, as shown in Equation (1):

(1) $$L_{MLM}(\theta ,\theta_{MLM})=-\sum_{i=1}^{M}\log P(token_{i}|\theta ,\theta_{MLM}).$$

In this equation, θ denotes the parameter of the Encoder part, $\theta_{MLM}$ is the parameter of the fully connected layer of the MLM task, M is the number of randomly masked tokens, and $token_{i}$ represents the token predicted by the model at position i of the sequence.

The mNSP task learns the matching relationship of payload by determining whether two sub-sequences belong to the same packet. Unlike natural language, encrypted traffic payloads are continuous byte streams without clear sentence demarcation or independent meaning, so it is not feasible to divide them using punctuation as in natural language. However, encrypted payloads from different plaintext have distinct ciphertext feature distributions, allowing for us to determine whether two sub-sequences belong to the same payload. In this task, payloads are divided into two nearly equal-length sub-sequences, each ending with [SEP] to mark the end of the sub-sequence. The second sub-sequence is replaced with another packet’s sub-sequence in 50% of cases. The complete input byte sequence consists of [CLS] at the beginning followed by the two sub-sequences. The output hidden layer vector corresponding to [CLS] is passed through a binary classifier to determine whether the sub-sequences belong to the same packet. This classifier comprises two fully connected layers. The negative log-likelihood function is used as the loss function for this task, as indicated in Equation (2):

(2) $$L_{mNSP}(\theta ,\theta_{mNSP})=-\sum_{j=1}^{N}\log P(y_{j}|\theta ,\theta_{mNSP}).$$

In this equation, $\theta_{mNSP}$ is the binary classifier parameter followed by Encoder, N is the number of input sequences, $y_{j}\in [0,1]$ is the output result of the binary classifier (1 represents paired sub-sequences and 0 represents unpaired ones).

The sum of the loss functions from both tasks is used to calculate the model loss for gradient updates, as shown in Equation (3):

(3) $$L(\theta ,\theta_{MLM},\theta_{mNSP})=L_{MLM}(\theta ,\theta_{MLM})+L_{mNSP}(\theta ,\theta_{mNSP}).$$

### 3.4. Fine-Tuning

During the fine-tuning phase, the model is trained on small-scale labeled datasets for given tasks to learn task-specific representations. Adjusting all parameters in a pre-trained model enables quick adaptation to downstream tasks. However, full parameter fine-tuning in a pre-trained model demands high computational and memory resources. As the model size and number of tasks increase, training and storing a new model for each task exacerbates the issue of inefficient parameter use.

The PEFT method, particularly LoRA [20], effectively mitigates this problem. We apply LoRA’s parallel matrix approach to the encrypted traffic classification model to conserve computational parameters. The advancement of LoRA over other PEFT methods in encrypted traffic classification is shown in Section 4.6.

LoRA involves inserting matrices parallel to the pre-trained model matrices while freezing the pre-trained model. This allows for the model to maintain new small-scale parameter matrices for downstream tasks. Unlike classic fine-tuning methods, the model can switch PEFT module parameters as needed for different traffic classification tasks without replacing the entire model’s parameters, enhancing parameter utilization efficiency and reducing deployment and switching costs for multi-task models. Compared to other PEFT methods, LoRA modules compute in parallel with the pre-trained model, avoiding the computational bottlenecks and inference delays of new serial modules.

LoRA updates only the query and value projection weight matrix of each Encoder’s multi-head attention layer and the final fully connected classifier layer. Specifically, leveraging the low “intrinsic rank” of over-parametrized models [20], it employs the product of two low-rank matrices to replace the original matrix for gradient updates during backpropagation, as demonstrated in Equation (4).

(4) $$h=W^{\left(i\right)}x+\Delta Wx=W^{\left(i\right)}x+\frac{\alpha }{r}BAx.$$

In this equation, $W^{\left(i\right)}\in {\mathbb{R}}^{d\times k}$ represents the projection weight matrix of layer i with rank r (r<<d,k) selected, the product of matrices $A\in {\mathbb{R}}^{r\times k}$, $B\in {\mathbb{R}}^{d\times r}$ is used to replace the updated weight matrix ΔW of $W^{\left(i\right)}$, x is the input vector, h is the output vector, and α is the deflation parameter of the updated weight matrix. We use a random Gaussian initialization for each element of A and zero for B. $W^{\left(i\right)}$ is kept frozen during the training process, and gradients are computed and updated for A, B.

**Figure 3:**
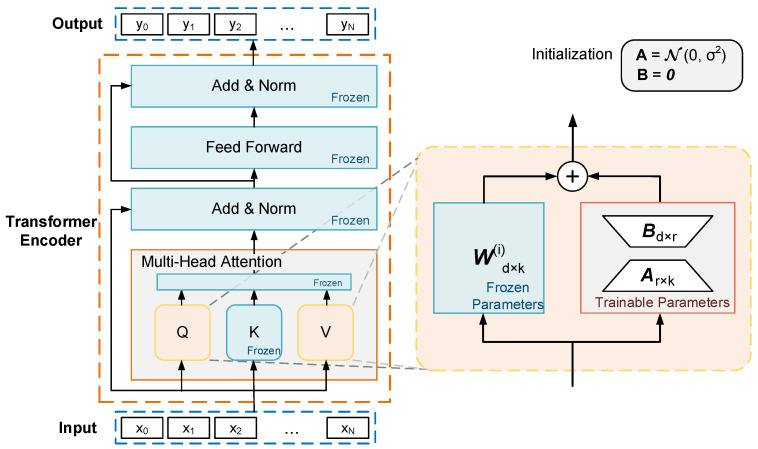
Fine-tuning computational diagram.

Drawing on the computational ideas of LoRA, we introduce this computational approach into our model. Figure 3 illustrates the computational operation for a single Encoder.

As for the fully connected classifiers, the classic LoRA method preserves the first fully connected layer parameter of the pre-trained model’s NSP task classifier and allows for only the second fully connected layer parameter to undergo gradient updating due to the consideration of the relevance of the NLP fine-tuning task to the pre-training task. However, because of the distinct association manner of encrypted traffic payloads compared to natural language sentences, continuing to use the first fully connected layer might not effectively synthesize feature information. Since the association between encrypted traffic payloads and natural language sentences differs, and different encryption algorithms encrypt traffic with varying byte-level features, to accommodate these differences and improve the model’s effectiveness, we design both fully connected layers of the classifier subjected to gradient updates.

The loss function for the fine-tuning stage is formulated as shown in Equation (5).

(5) $$L(\theta_{A,B},\theta_{cls})=-\sum_{i=1}^{k}\log P(predict_{i}|\theta ,\theta_{A,B},\theta_{cls}).$$

Here, $\theta_{A,\;B}$ represent the parameters of all A and B, $\theta_{cls}$ is the classifier, θ is the frozen parameters from the pre-trained model, k is the batch, and $predict_{i}$ is the classifier’s prediction label.

## 4. Experiments

This section validates the advanced performance of the PETReLM from multiple perspectives. We first introduce the datasets, experimental setup, and evaluation metrics used in our experiments, then present the classification results of the model on these datasets, and follow by ablation studies to prove the effectiveness of each module.

### 4.1. Datasets

For model pre-training and fine-tuning, we utilize various public encrypted traffic datasets, each with its specific characteristics:

Browser2020 [4]: This dataset comprises traffic generated by accessing the top 1000 websites on Alexa using four different browsers: Google, Firefox, Samsung Internet, and UC. Each website visit lasts for 15 s, with scripts simulating random clicks and browsing behavior.

CIC-IDS 2017 [34]: A network attack traffic dataset containing benign and malicious traffic based on protocols like HTTP, HTTPS, FTP, and SSH. The traffic is segmented into different time periods, each producing various types of traffic.

ISCXTor2017 [35]: A dataset focusing on The Onion Routing (Tor) traffic which includes eight different types of Tor traffic collected using onion routing.

ISCXVPN2016 [36]: A Virtual Private Network (VPN) traffic dataset that gathers traffic generated by different types of applications under conditions of using or not using a VPN, simulated between two hosts. This dataset is one of the most commonly used datasets currently. The dataset categorizes encrypted applications into 17 classes and encrypted services into 12 classes.

USTC-TFC2016 [37]: This dataset comprises malicious traffic collected in a real network environment alongside normal traffic.

CIC IoT Dataset 2023 [38]: This dataset contains IoT attack traffic collected from a topology consisting of 105 IoT devices. It includes both normal IoT traffic and network attack traffic generated by 33 types of malicious IoT devices.

The granularity of encrypted traffic classification mainly includes a packet level and a session level. Encrypted traffic sessions contain more information than individual packets. However, it is challenging to obtain datasets with sufficient diversity and undisputed ground truth [19]. Labeled encrypted traffic datasets may suffer from class sample imbalances, with limited samples collected for certain categories under constrained conditions. For instance, in the ISCXVPN2016 dataset (see Table 2), the AIM and ICQ categories each contain only 49 and 45 valid sessions, respectively (we consider a session to be valid if it contains more than 4 packets). The limited samples may hinder deep learning models from effectively learning sample representations. Therefore, we confine our study to packet-level representation learning. We validate that valuable information about traffic can still be obtained from packets. Additionally, packet-level traffic classification can alleviate the problem of insufficiently labeled training samples.

For pre-training, we use benign traffic from Browser, CIC-IDS 2017, and the CIC IoT Dataset 2023 as the pre-training dataset. This dataset comprises 955,000 unlabeled traffic data, totaling 11.3 GB. For fine-tuning, we use datasets as shown in Table 2. It is worth mentioning that our datasets encompass a wide range of protocols such as QUIC. By selecting datasets that cover a variety of protocols, we aim to enable the model to learn more general and effective representations. We select datasets from various classification scenarios to validate the model’s performance. ISCXT8, ISCXS12, and ISCXA17 simulate traffic classification in network management scenarios, while USTC20 and CICIoT33 simulate malicious traffic classification in network security scenarios. Our experiment ensures the orthogonality between the pre-trained and fine-tuned datasets, simulating the scenario where the model learns representations of encrypted traffic it has never seen before.

We select 5000 samples from each category in the fine-tuning dataset with a ratio of 8:1:1 for the training, validation, and test sets, respectively.

### 4.2. Experimental Settings

The experiments are conducted using the NVIDIA Tesla V100 GPU, with Python version 3.10.12, CUDA version 11.7, and PyTorch version 1.13.0.

In BERT-base, each multi-head self-attention sublayer within the Encoders contains 12 attention heads. The dimension of the embedding vectors is set to 768, and the maximum length for input vector sequences is 512.

During the model’s pre-training phase, the batch size is set to 32, with a total step count of 500,000 and a learning rate of 2 × 10^−5^. For the fine-tuning phase, the batch size remains at 32, and the learning rate is adjusted to 8 × 10^−4^. We consistently use AdamW as the optimization tool. The deflation parameter of the updated weight matrix is set to 32, and the rank for the weight update matrix is set to 4. The fine-tuning process is conducted over 10 epochs.

**Figure 4:**
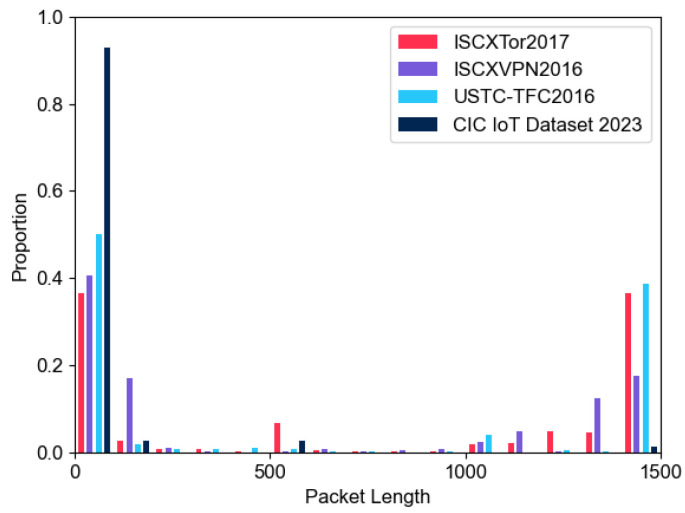
The network layer load length distribution of fine-tunning datasets.

By comparing the distribution of network layer payload lengths in each fine-tuning dataset, we determine the specific truncation length. Figure 4 illustrates the distribution of network layer payload lengths for the four fine-tuning datasets. We observe that the payload distribution of the datasets mostly falls below 300 bytes, with some datasets having a proportion of over 1000 bytes. As shown in Figure 2, since most byte pairs are converted into one token and a byte appears in two adjacent byte pairs, it is reasonable to approximate one byte as one token. If a larger value is chosen for the input token length, a high proportion of samples requires padding with [PAD]. Conversely, selecting a smaller value compromises the model’s ability to learn representations of the application layer payload. Considering the overall distribution of dataset payloads and the generality of the pre-trained model, we opt to use 512 tokens of the payload as the maximum input length for pre-training. With TCP packets having a fixed header of 20 bytes and UDP packets having an 8-byte header, this choice ensures the retention of complete transport layer header information and most of the application layer encrypted payload while mitigating the impact of excessive [PAD] on model classification results.

### 4.3. Evaluation Metrics

We use classic metrics to evaluate model performance, including Accuracy (Acc), Precision (Pre), Recall (Rec), and F1-Score (F1). For binary classification problems, the equations are as follows:

(6) $$\mathrm{Acc}=\frac{\mathrm{TP}+\mathrm{TN}}{\mathrm{TP}+\mathrm{FP}+\mathrm{FN}+\mathrm{TN}}$$

(7) $$\mathrm{Pre}=\frac{\mathrm{TP}}{\mathrm{TP}+\mathrm{FP}}$$

(8) $$\mathrm{Rec}=\frac{\mathrm{TP}}{\mathrm{TP}+\mathrm{FN}}$$

(9) $$F1=2\times \frac{\mathrm{Precision}\times \mathrm{Recall}}{\mathrm{Precision}+\mathrm{Recall}}$$

where TP represents true positives, FP is false positives, TN is true negatives, and FN is false negatives. In multi-classification scenarios, we adopt the Macro Average [39] method to calculate Precision, Recall, and F1-Score. It involves calculating these metrics for each category and then averaging the results. The accuracy metric is not affected by the multi-class nature of the problem.

### 4.4. Performance Analysis

PETReLM’s performance is compared against baseline models based on deep learning including 1D-CNN [11], Deep Packet [10], PERT [13], and ET-BERT [14]. The motivation for selecting these baseline models is that they represent typical deep learning methods applied to encrypted traffic classification. Comparing against these baseline models can objectively demonstrate the performance of our model. These models are replicated with their original structure and parameters, and their performance (in terms of Acc, F1) is compared with our model across various datasets, as shown in Table 3.

Based on the experimental results, we observe that individual packets indeed contain sufficient representations for distinguishing between traffic categories. Most methods are effective in accurately classifying designed scenarios based on packet representations. Furthermore, convolutional deep learning methods (1D-CNN, Deep Packet) exhibit unstable performance: they show significant discrepancies in performance on USTC20 and CICIoT33 datasets. There are two main reasons: (a) Convolutional neural networks have a relatively narrow low-level field of view. Convolutions capture relationships between nearby bytes, while distant relationships can only be learned at higher layers. Therefore, the overall byte relationship extraction capability of convolutional methods may be lacking, resulting in inferior representation extraction performance on complex datasets. (b) Convolutional neural networks lack prior knowledge of encrypted traffic. Un-pretrained convolutional methods cannot acquire universal representations of traffic and only learn proprietary representations in limited datasets. The lack of incremental learning may also lead to subpar performance.

In contrast, the excellent performance of pretraining-based methods highlights the strong appeal of deep learning frameworks for encrypted traffic classification. Pretraining models with large-scale parameters and architectures conducive for learning long sequences are more suitable for encrypted traffic classification tasks. Specifically, ET-BERT and PERT have similar structures and training methods. PERT uses the ALBERT architecture to share all parameters between layers, resulting in performance fluctuations observed in the ISCXA17 dataset. ET-BERT’s performance declines on the CICIoT33 dataset. Overall, the PETReLM demonstrates more balanced performance across different application scenarios, and it also performs similarly to the best baseline models.

**Figure 5:**
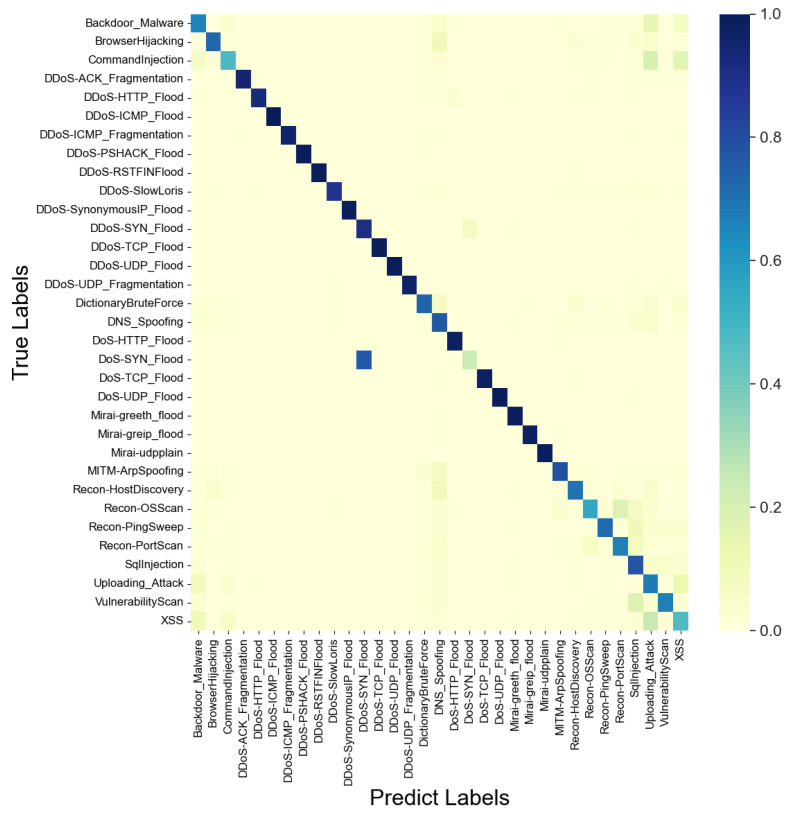
Classification heatmap of IoT attack.

At the same time, we identify potential limitations in our approach. From the classification results, we observe that the effectiveness of classifying IoT attack scenarios is notably lower compared to other scenarios. As illustrated in Figure 5, the PETReLM classification heatmap reveals that the model nearly classifies all DoS SYN Flood traffic as DDoS SYN Flood. This discrepancy arises because the primary difference between the two attacks lies in the flood attack originating from multiple or fewer source hosts, which may not be clearly evident at the level of individual packets. Similar issues are observed with Command Injection, Uploading Attacks, and XSS, where all attacks involve similar data transmission methods such as Web requests, and their application layer payloads may also be comparable. These challenges extend to other confused categories, where the representations of these categories within a single packet are similar, making them difficult to distinguish. In summary, we find that the limitations of this approach may arise in scenarios where the representation of individual packet is not sufficiently clear, and relying solely on packet-level information may not effectively classify data accurately.

### 4.5. Resource Usage Analysis

To compare resource usage, we take the USTC20 task as an example. We compare the parameter scales of all baseline methods and also examine the GPU usage of pre-training models.

Table 4 reveals that settings of convolution-based methods affect the parameter scale, with an average magnitude of around 10^5^. Pre-training methods have parameters scaled 1–2 orders of magnitude higher than convolutional methods. PERT reduces the parameter scale by sharing parameters between layers, but fluctuates in performance. The PETReLM reduces the parameter scale by inserting additional modules while maintaining stable performance. PETReLM’s parameter scale accounts for only 5.6% and 21.0% of ET-BERT and PERT, respectively, and is in the same order of magnitude as small-scale convolutional models. The PETReLM utilizes the least GPU memory among pretrained models because only a small fraction of parameters accepts gradient updates, reducing GPU resource usage for storing gradients. Updating only a small fraction of parameters also accelerates model computation speed, reducing training time by 16.68% compared to ET-BERT. The PETReLM is more suitable for multitask applications, as conventional methods require storing all model parameters for each task, while the PETReLM only needs to store the inserted modules to preserve task-specific feature extraction capabilities. When task switching is needed, the PETReLM can quickly switch between different task scenarios by only switching small modules.

Overall, the PETReLM ensures that the model’s complexity is sufficient to learn the basic representations of encrypted traffic while significantly reducing the required training resources. It facilitates rapid task switching in multitask scenarios while maintaining classification performance comparable to that of state-of-art models.

### 4.6. Ablation Analysis

To validate the effectiveness of our fine-tuning approach, we conduct ablation experiments focusing on two aspects:

(1) Suitability of the modified LoRA for fine-tuning pre-trained models in encrypted traffic classification. We compare it with other traditional PEFT methods like Adapter [22], Prefix Tuning [21], P-Tuning [25], and IA3 [24].
(2) Applicability of using two fully connected layers as a classifier for encrypted traffic classification. We compare two approaches: (i) Using a classifier composed of two fully connected layers, where the first layer parameters from the mNSP task are preserved and the second layer is subject to gradient updates (mNSP1 + FC2); (ii) Using a classifier composed solely of one fully connected layer, which reduces the input dimension to the classification dimension (FC1).

The experiments are conducted on the USTC20 dataset, and the results are presented in Table 5.

The findings indicate that compared to other PEFT methods and classifier configurations, our model achieves superior results in encrypted traffic classification tasks. The Adapter method is similar to the PETReLM in that both insert modules that function to downscale and then upscale the input vectors to the output. The main difference is that Adapter inserts serial parameter modules between layers while the PETReLM inserts parallel parameter modules in the layers. IA3 reduces trainable parameters through vector-scaled activation. Prefix Tuning and P-Tuning construct prompts to minimize fine-tuning parameters. The PETReLM shows improvement in F1-Score compared to these methods. When comparing different classifier configurations, performance slightly drops with both the original classifier and the use of a single fully connected layer as a classifier. Notably, even with a 75% reduction in trainable parameter volume when using only one fully connected layer, the performance exhibits only minor fluctuations. Therefore, employing a single fully connected layer as a classifier also emerges as a balanced choice between performance and resource utilization.

## 5. Interpretability Analysis

This section analyzes the model’s matrix parameters using singular value decomposition, comparing the changes between pre-trained and fine-tuned model parameters to explain the mechanism of learning traffic representations. We use the original pre-trained model, the fully fine-tuned model, and the PETReLM for interpretability analysis, aiming to explain the underlying learning mechanisms of the full fine-tuning and PEFT methods by analyzing the correlations between model parameters in the pre-training and fine-tuning phases.

### 5.1. Singular Value Analysis

The magnitude of the singular value corresponds to the importance of the left and right singular vectors in a matrix. A large singular value indicates primary structures and directions represented by their associated vectors. Singular values and singular vectors are primarily used for interpretability analysis. In this section, we take the ISCXA17 task as an example, and set $W_{p}^{\left(i\right)}$, $W_{f}^{\left(i\right)}$, and $W_{m}^{\left(i\right)}$ to represent the query projection matrices of the multi-head self-attention mechanism of the Encoder at layer i of the pre-trained model (*p*), the fully fine-tuned model (*f*), and the PETReLM (*m*), respectively. We merge PETReLM’s parallel module with the pre-trained model to align the model sizes. The singular value decomposition of matrix $W_{p}^{\left(i\right)}$ is as shown in Equation (10):

(10) $$W_{p}^{\left(i\right)}=U_{p}^{\left(i\right)}\Sigma_{p}^{\left(i\right)}{\left(V_{p}^{\left(i\right)}\right)}^{T}.$$

Setting the diagonal element $\sigma_{p}^{\left(i,j\right)}$ represents the jth singular value of $\Sigma_{p}^{\left(i\right)}$. We define the magnification factor of the fine-tuned matrix’s *j*th singular value as the ratio of the *j*th singular value of the fine-tuned matrix to the *j*th singular value of the pre-trained matrix. For instance, the calculation formula of the *j*th singular value magnification factor for the fully fine-tuned model is shown in (11):

(11) $$f\left(W_{f}^{\left(i\right)},W_{p}^{\left(i\right)},j\right)=\frac{\sigma_{f}^{\left(i,j\right)}}{\sigma_{p}^{\left(i,j\right)}}.$$

**Figure 6:**
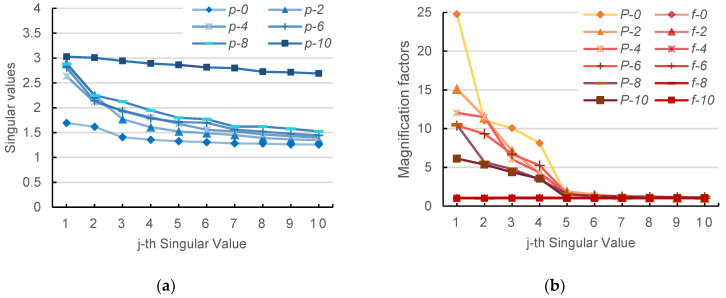
Comparison of singular values between pre-trained and fine-tuned models. (**a**) Pre-trained model singular values of different layers; (**b**) Magnification factors of different fine-tuned matrix’s singular values.

Figure 6a displays the *j*th singular value of $W_{p}^{\left(i\right)}$ for different values of *i* and *j* (represented as *p*-*i* in the figure), while Figure 6b shows the magnification factors (represented as *f*-*i*, *P*-*i* in the figure) for $W_{f}^{\left(i\right)}$ and $W_{m}^{\left(i\right)}$. For brevity, only the first 10 singular values of the odd-numbered layers are shown in the figures.

It can be observed that the singular values of the fully fine-tuned model and the pre-trained model are closely aligned, increasing with layer number i, but they are generally small in value. PETReLM significantly amplifies the first four singular values. Similar results are observed in the value projection matrix and other matrices of the fully fine-tuned model.

Overall, the main features of the first layer query projection matrix of the pre-trained model and the fully fine-tuned model are not pronounced, while the main features, especially in the last two layers near the output, are more prominent. The PETReLM amplifies certain features of the original matrices to varying degrees. The magnification of singular values in lower layers by the PETReLM is significantly higher than in higher layers, indicating substantial changes made by the PETReLM to the pre-trained model. The extent of these changes is greater in extracting basic representations in lower layers compared to abstract representations in higher layers.

### 5.2. Subspace Similarity Analysis

To analyze the adjustments made by the fine-tuned model to the parameters of the pre-trained model, we compare the feature and structural similarity of matrices. We measure the similarity by comparing the overlap of the subspaces formed by the first k∈[1,784] right singular vectors of the query projection matrix in the first-layer Encoder of the pre-trained model and the fine-tuned model. The normalized matrix similarity [40] is used to calculate the subspace similarity, as shown in (12):

(12) $$d\left(A,B\right)=\frac{\sum_{i=1}^{p}{\sigma_{i}}^{2}}{p}\;p=\min\left\{m,n\right\}.$$

Here, $A\in {\mathbb{R}}^{l\times n}$ and $B\in {\mathbb{R}}^{l\times n}$ represent matrices composed of the first m and n l-dimensional right singular vectors, respectively, with $\sigma_{i}$ being the *i*th singular value of matrix $A^{T}B$. When the value of the normalized matrix similarity tends to one, it means that the subspaces formed by A, B have a high degree of overlap; equality to one means complete overlap; tendency to zero means low overlap; and equality to zero means no overlap at all.

**Figure 7:**
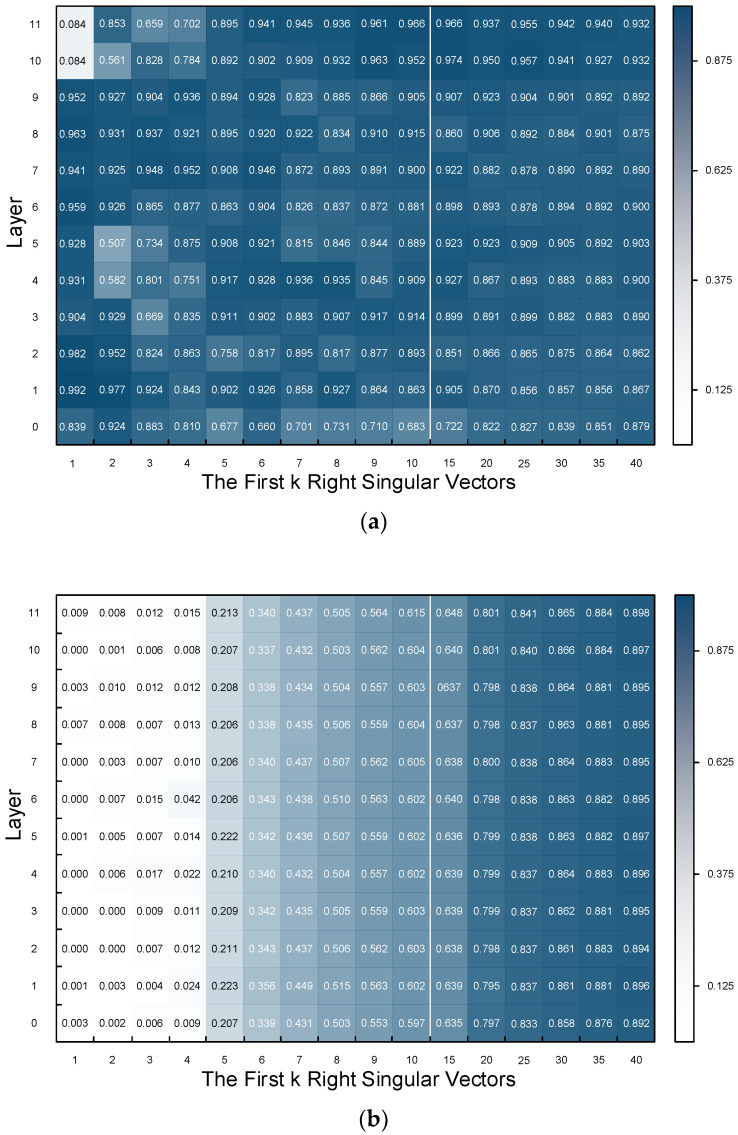
Subspace similarity. (**a**) Subspace similarity of the fully fine-tuned model with the pre-trained model; (**b**) Subspace similarity of the PETReLM with the pre-trained model.

Figure 7a,b shows the similarity of the subspaces formed by the first k right singular vectors of each layer of the pre-trained model with the fully fine-tuned model and the PETReLM, respectively.

Each square in the figure represents the similarity of the subspace consisting of the first k vectors of the matrices. The first 40 singular vectors already illustrate the pattern well. The figure reveals that, apart from some fluctuations, the subspace similarity of the fully fine-tuned model with the pre-trained model remains at a high level. The subspace formed by the right singular vectors corresponding to the largest four singular values of the PETReLM and the pre-trained model barely overlap. However, as the dimension number of the subspace increases, the similarity rapidly grows, with most of the subspace formed by the first 25 singular values overlapping. Each layer shows the same trend. Similar results are observed for the left singular vectors.

### 5.3. Summary of Interpretability Findings

Combining the changes in singular values and the subspace similarity between model matrices, we observe that the structure and features of the pre-trained model’s query projection matrices are balanced and smooth. The matrices in the lowest layer are the smoothest, while the structure and features of the matrices in the higher layers are relatively more prominent, with the middle layers having fewer prominent features. This characteristic is more suitable for extracting general representations of traffic: the lower layers use smoother matrices to extract general byte representations of the payload, the middle layers continually filter and process representations from byte representations to abstract features, and the higher layers use matrices with more pronounced features to extract abstract features.

The fully fine-tuned model essentially maintains the structure and features of the pre-trained model’s matrices, making only minor adjustments to the matrix structure. However, since full fine-tuning alters the parameters of each matrix in the model, it is challenging to observe the actual changes made by fine-tuning.

The PETReLM, by updating a minimal number of matrix parameters instead of the full parameter set, allows for us to capture the subtle parameter changes at each layer, reflecting the fine-tuning adjustments made to the parameters of the pre-trained model. The PETReLM alters the model’s most crucial structure and features of the pre-trained model while maintaining the essential structure and features. Specifically, at the microscopic level, the matrix does not replicate the primary feature directions of the original matrix but instead amplifies directions not emphasized in the original matrix. The amplification is greater in the lower layers than in the higher layers. Macroscopically, this is reflected in the rapid increase in subspace similarity within a certain range as the value of k increases. Therefore, fine-tuning alters the overall feature extraction framework of the pre-trained model, thoroughly changing the primary feature extraction pattern at each layer. This includes more significant adjustments in the lower layers near the input for byte feature extraction and relatively minor macroscopic changes in the higher layers for abstract feature processing. This approach is more suitable for extracting specific traffic representations for a given classification task, with the lower layers filtering and retaining representation capabilities relevant to the given task and the higher layers focusing more on processing abstract features related to the task.

## 6. Conclusions

Motivated by the need to achieve encrypted traffic functional service classification under limited conditions, we propose the PEFT-based encrypted traffic classification method, the PETReLM. The method utilizes low-rank matrices instead of weight update matrices for parameter fine-tuning, enabling precise functional classification at the packet level for downstream tasks. We evaluate PETReLM’s generalization, robustness, and effectiveness across various network analysis and network security scenarios, including Tor service classification, VPN network service classification, VPN application classification, malware classification, and IoT attack traffic classification. When compared with existing methods, PETReLM maintains performance comparable to that of advanced models while reducing the trainable parameters by 99.44%, effectively saving computational and storage resources and reducing the deployment and switching costs of multi-task models. We explain the underlying principles and mathematical logic of how the PETReLM extracts features from the encrypted traffic, representing a novel attempt in encrypted traffic classification. We find that the PETReLM changes the characteristics of model parameters so that parameter hierarchy becomes distinct and the structure remains basically the same, which enhances the interpretability of the model.

However, we also identify some limitations in our approach. Our method does not consistently outperform existing state-of-the-art models in experimental performance. Additionally, packet-level identification introduces inherent limitations to the classification model. In scenarios where packet-level representations are not sufficiently effective, the classification results may not be optimal. Therefore, designing a universal encrypted traffic classification model may be more suitable for benign network environments in the field of network application detection. For environments where malicious or deceptive traffic may exist, more targeted designs are needed.

In the future, we hope to combine neural network model architectures with encrypted traffic algorithms to analyze the representation extraction mechanisms of encrypted traffic, further improving model classification effectiveness, and providing a more universally effective interpretative approach for encrypted traffic classification.

## Figures and Tables

**Table 1:** Comparison of encrypted traffic classification algorithms.

Input Data	Algorithms	Paper
IP, Port, Packet Lenth, Packet Direction, TLS Certificate	Random Forest, Correlation Graph	FlowPrint [4]
IP, Protocol, Packet Length	Random Forest	APPScanner [5]
Packet Lenth, Packet Direction	Random Forest	Conti et al. [6]
Certificate, Packet Length	Second-order Markov Chains	Shen et al. [7]
Packet Length, Arrival Time	Auto Encoder	Yu et al. [8]
Packet Lenth, Packet Direction	GNN	Shen et al. [9]
Payload	1D-CNN	Wang et al. [11]
Payload	SAE, 1D-CNN	Deep Packet [10]
Payload	1D-CNN, BiLSTM	TCSRNN [2]
Payload	BiLSTM, TextCNN	Jiang et al. [12]
Payload	ALBERT	PERT [13]
Payload	BERT	ET-BERT [14]

**Table 2:** Description of fine-tuning datasets.

Dataset	Task	Classes	Classification Basis	Specific Categories
ISCXTor2017 [35]	ISCXT8	8	Tor Service	Audio, Browsing, Chat, FTP, Mail, P2P, Video, VoIP
ISCXVPN2016 [36]	ISCXS12	12	VPN Service	VPN: Email, Chat, Stream, File transfer, VoIP, P2P; Non-VPN: Email, Chat, Stream, File transfer, VoIP, P2P
	ISCXA17	17	VPN Application	AIM, Email, Facebook, FTP, Gmail, Hangouts, ICQ, Netflix, P2P, SCP, SFTP, Skype, Spotify, tor, Vimeo, Voipbuster, Youtube
USTC-TFC2016 [37]	USTC20	20	Malware	Benign: BitTorrent, Facetime, FTP, Gmail, MySQL, Outlook, Skype, SMB, Weibo, World of Warcraft; Malware: Cridex, Geodo, Htbot, Miuref, Neris, Nsis-ay, Shifu, Tinba, Virut, Zeus
CIC IoT Dataset 2023 [38]	CICIoT33	33	IoT Cyberattack	DDoS: ACK Fragmentation, HTTP Flood, ICMP Flood, ICMP Fragmentation, PSHACK Flood, RSTFIN Flood, SlowLoris, SynonymousIP Flood, SYN Flood, TCP Flood, UDP Flood, UDP Fragmentation; Brute Force: Dictionary Brute Force; Spoofing: ARP Spoofing, DNS Spoofing; DoS: HTTP Flood, SYN Flood, TCP Flood, UDP Flood; Recon: Host Discovery, OS Scan, Ping Sweep, Port Scan, Vulnerability Scan; Web Based: Backdoor Malware, Browser Hijacking, Command Injection, SQL Injection, Uploading Attack, XSS; Mirai: Greeth Flood, GREIP flood, UDPPlain

**Table 3:** Comparative experiment results of different methods.

Model	ISCXT8		ISCXS12		ISCXA17		USTC20		CICIoT33	
	Acc	F1	Acc	F1	Acc	F1	Acc	F1	Acc	F1
1D-CNN [11]	0.9704	0.9709	0.9149	0.9147	0.9265	0.9181	0.7738	0.7508	0.7280	0.7307
Deep Packet [10]	0.9722	0.9727	0.9169	0.9153	0.9154	0.9073	0.7911	0.7659	0.6987	0.7029
PERT [13]	0.9995	0.9995	0.9705	0.9706	0.9816	0.9782	0.9875	0.9876	0.8419	0.8400
ET-BERT [14]	0.9998	0.9997	0.9785	0.9786	0.9911	0.9893	0.9940	0.9940	0.8065	0.8094
PETReLM	0.9998	0.9997	0.9685	0.9686	0.9867	0.9830	0.9881	0.9881	0.8234	0.8247

**Table 4:** Parameter scales of different methods.

	Trainable Parameters	Pretraining Parameters	Percentage (%)	GPU (GB)
1D-CNN [11]	5.7 × 10^6^	-	-	-
Deep Packet [10]	10.1 × 10^6^	-	-	-
PERT [13]	36.7 × 10^6^	36.7 × 10^6^	100	24.8
ET-BERT [14]	136.3 × 10^6^	136.3 × 10^6^	100	23.3
PETReLM	7.7 × 10^6^	136.3 × 10^6^	5.6	19.3

**Table 5:** Comparison of ablation results.

Method	Acc	Pre	Rec	F1
Adapter	0.9839	0.9842	0.9839	0.9839
Prefix Tuning	0.9706	0.9713	0.9706	0.9702
P-Tuning	0.8734	0.8816	0.8734	0.8722
IA3	0.9698	0.9703	0.9698	0.9695
mNSP1 + FC2	0.9824	0.9824	0.9824	0.9817
FC1	0.9861	0.9863	0.9861	0.9862
PETReLM	0.9878	0.9888	0.9878	0.9877

## Data Availability

Data are contained within the article.

## References

[1] Isingizwe D.F., Wang M., Liu W., Wang D., Wu T., Li J. Analyzing Learning-Based Encrypted Malware Traffic Classification with AutoML. Proceedings of the 2021 IEEE 21st International Conference on Communication Technology (ICCT).

[2] Lin K., Xu X., Gao H. (2021). TSCRNN: A Novel Classification Scheme of Encrypted Traffic Based on Flow Spatiotemporal Features for Efficient Management of IIoT. Comput. Netw..

[3] Papadogiannaki E., Ioannidis S. (2021). A survey on encrypted network traffic analysis applications, techniques, and countermeasures. ACM Comput. Surv. (CSUR).

[4] Van Ede T., Bortolameotti R., Continella A., Ren J., Dubois D.J., Lindorfer M., Choffnes D., Van Steen M., Peter A. (2020). FlowPrint: Semi-Supervised Mobile-App Fingerprinting on Encrypted Network Traffic. Network and Distributed System Security Symposium.

[5] Taylor V.F., Spolaor R., Conti M., Martinovic I. (2017). Robust smartphone app identification via encrypted network traffic analysis. IEEE Trans. Inf. Forensics Secur..

[6] Conti M., Mancini L.V., Spolaor R., Verde N.V. (2016). Analyzing Android Encrypted Network Traffic to Identify User Actions. IEEE Trans. Inform. Forensic Secur..

[7] Shen M., Wei M., Zhu L., Wang M. (2017). Classification of Encrypted Traffic With Second-Order Markov Chains and Application Attribute Bigrams. IEEE Trans. Inform. Forensic Secur..

[8] Yu T., Zou F., Li L., Yi P. An Encrypted Malicious Traffic Detection System Based on Neural Network. Proceedings of the 2019 International Conference on Cyber-Enabled Distributed Computing and Knowledge Discovery (CyberC).

[9] Shen M., Zhang J., Zhu L., Xu K., Du X. (2021). Accurate Decentralized Application Identification via Encrypted Traffic Analysis Using Graph Neural Networks. IEEE Trans. Inform. Forensic Secur..

[10] Lotfollahi M., Jafari Siavoshani M., Shirali Hossein Zade R., Saberian M. (2020). Deep Packet: A Novel Approach for Encrypted Traffic Classification Using Deep Learning. Soft Comput..

[11] Wang W., Zhu M., Wang J., Zeng X., Yang Z. End-to-End Encrypted Traffic Classification with One-Dimensional Convolution Neural Networks. Proceedings of the 2017 IEEE International Conference on Intelligence and Security Informatics (ISI).

[12] Jiang T., Yin W., Cai B., Zhang K. (2021). Encrypted malicious traffic identification based on hierarchical spatiotemporal feature and multi-head attention. Comput. Eng..

[13] He H., Yang Z., Chen X. PERT: Payload Encoding Representation from Transformer for Encrypted Traffic Classification. Proceedings of the 2020 ITU Kaleidoscope: Industry-Driven Digital Transformation (ITU K).

[14] Lin X., Xiong G., Gou G., Li Z., Shi J., Yu J. ET-BERT: A Contextualized Datagram Representation with Pre-Training Transformers for Encrypted Traffic Classification. Proceedings of the ACM Web Conference 2022.

[15] Aceto G., Ciuonzo D., Montieri A., Pescapè A. (2021). DISTILLER: Encrypted traffic classification via multimodal multitask deep learning. J. Netw. Comput. Appl..

[16] Wang P., Chen X., Ye F., Sun Z. (2019). A survey of techniques for mobile service encrypted traffic classification using deep learning. IEEE Access.

[17] Chen Z., Cheng G., Xu Z. (2023). A survey on Internet encrypted traffic detection classification and identification. Chin. J. Comput..

[18] Devlin J., Chang M., Lee K., Toutanova K. BERT: Pre-training of Deep Bidirectional Transformers for Language Understanding. Proceedings of the Conference of the North American Chapter of the Association for Computational Linguistics (NAACL).

[19] Shen M., Ye K., Liu X., Zhu L., Kang J., Yu S., Li Q., Xu K. (2023). Machine Learning-Powered Encrypted Network Traffic Analysis: A Comprehensive Survey. IEEE Commun. Surv. Tutor..

[20] Hu E.J., Shen Y., Wallis P., Allen-Zhu Z., Li Y., Wang S., Wang L., Chen W. (2021). Lora: Low-rank adaptation of large language models. arXiv.

[21] Kang P., Yang H., Ma H. (2022). TLS Malicious Encrypted Traffic Identification Research. J. Comput. Eng. Appl..

[22] Gehring J., Miao Y., Metze F., Waibel A. Extracting Deep Bottleneck Features Using Stacked Auto-Encoders. Proceedings of the 2013 IEEE International Conference on Acoustics, Speech and Signal Processing.

[23] Huang Z., Xu W., Yu K. (2015). Bidirectional LSTM-CRF models for sequence tagging. arXiv.

[24] Kim Y. (2014). Convolutional neural networks for sentence classification. arXiv.

[25] Lan Z., Chen M., Goodman S., Gimpel K., Sharma P., Soricut R. (2019). ALBERT: A Lite BERT for Self-supervised Learning of Language Representations. arXiv.

[26] Pfeiffer J., Kamath A., Rücklé A., Cho K., Gurevych I. (2020). AdapterFusion: Non-destructive task composition for transfer learning. arXiv.

[27] Li X.L., Liang P. (2021). Prefix-Tuning: Optimizing Continuous Prompts for Generation. arXiv.

[28] Houlsby N., Giurgiu A., Jastrzebski S., Morrone B., de Laroussilhe Q., Gesmundo A., Attariyan M., Gelly S. Parameter-efficient transfer learning for nlp. Proceedings of the International Conference on Machine Learning (ICML).

[29] Vaswani A., Shazeer N., Parmar N., Uszkoreit J., Jones L., Gomez A., Kaiser L., Polosukhin I. (2017). Attention is all you need. Adv. Neural Inf. Process. Syst..

[30] Liu H., Tam D., Muqeeth M., Mohta J., Huang T., Bansal M., Raffel C. Few-Shot Parameter-Efficient Fine-Tuning is Better and Cheaper than In-Context Learning. Proceedings of the NeurIPS.

[31] Liu X., Zheng Y., Du Z., Ding M., Qian Y., Yang Z., Tang J. (2021). GPT understands, too. arXiv.

[32] Wu Y., Schuster M., Chen Z., Le Q.V., Norouzi M., Macherey W., Krikun M., Cao Y., Gao Q., Macherey K. (2016). Google’sneural machine translation system: Bridging the Gap between human and machine translation. arXiv.

[33] Doğanaksoy A., Ege B., Koc¸ak O., Sulak F. (2010). Cryptographic randomness testing of block ciphers and hash functions. Cryptol. Eprint Arch..

[34] Sharafaldin I., Habibi Lashkari A., Ghorbani A.A. (2018). Toward Generating a New Intrusion Detection Dataset and Intrusion Traffic Characterization: In International Conference on Information Systems Security and Privacy.

[35] Lashkari A., Draper Gil G., Mamun M.S.I., Ghorbani A.A. (2017). Characterization of Tor Traffic Using Time Based Features: In 3rd International Conference on Information Systems Security and Privacy.

[36] Draper-Gil G., Lashkari A.H., Mamun M.S.I., Ghorbani A.A. (2016). Characterization of Encrypted and VPN Traffic Using Time-Related Features: In 2nd International Conference on Information Systems Security and Privacy.

[37] Wang W., Zhu M., Zeng X., Ye X., Sheng Y. Malware Traffic Classification Using Convolutional Neural Network for Representation Learning. Proceedings of the 2017 International Conference on Information Networking (ICOIN).

[38] Neto E.C.P., Dadkhah S., Ferreira R., Zohourian A., Lu R., Ghorbani A.A. (2023). CICIoT2023: A Real-Time Dataset and Benchmark for Large-Scale Attacks in IoT Environment. Sensors.

[39] Liu C., Wang W., Wang M., Lv F., Konan M. (2017). An Efficient Instance Selection Algorithm to Reconstruct Training Set for Support Vector Machine. Knowl.-Based Syst..

[40] Hamm J., Lee D.D. Grassmann discriminant analysis: A unifying view on subspace-based learning. Proceedings of the 25th International Conference on Machine Learning.

